# Sleep in Juvenile Idiopathic Arthritis: An Exploratory Investigation of Heart Rate Variability

**DOI:** 10.3390/brainsci15060648

**Published:** 2025-06-17

**Authors:** M. C. Lopes, S. Roizenblatt, L. M. A. Soster, K. Spruyt

**Affiliations:** 1 Polisomnography Unit, Children’s Institute, Medical School, University of Sao Paulo, Sao Paulo 05360-160, Brazil; let.azevedo@gmail.com; 2 Childhood and Adolescence Affective Disorders Program (PRATA), Institute and Department of Psychiatry, University of Sao Paulo, Sao Paulo 05508-220, Brazil; 3 Clinical Medicine Discipline, Federal University of São Paulo (UNIFESP), Sao Paulo 04039-032, Brazil; suelyroi@gmail.com; 4 NeuroDiderot, L’Institut National de la Santé et de la Recherche Médicale (INSERM), Université Paris Cité, 75006 Paris, France; karen.spruyt@inserm.fr

**Keywords:** heart rate variability, sleep fragmentation, juvenile idiopathic arthritis

## Abstract

**Introduction:** The monitoring of autonomic nervous balance during childhood remains underexplored. However, heart rate variability (HRV) is widely recognized as a biomarker of health risk across the lifespan. Juvenile idiopathic arthritis (JIA), a group of chronic inflammatory joint disorders, is associated with persistent inflammation and pain, both of which contribute to increased cardiovascular risk, commonly linked to reduced HRV. Among HRV parameters, very-low frequency (VLF) components have been associated with physiological recovery processes. This study aimed to assess HRV during sleep in patients with JIA. **Methods:** We studied 10 patients with JIA and 10 age-, gender-, and Tanner stage-matched healthy controls. All participants underwent polysomnographic monitoring following an adaptation night in the sleep laboratory. HRV was analyzed using standard time and frequency domain measures over 5 min epochs across all sleep stages. Frequency components were classified into low- and high-frequency bands, and time domain measures included the standard deviation of the beat-to-beat intervals. Group differences in HRV parameters were assessed using nonparametric tests for independent samples, with a significance level set at *p* < 0.05. **Results:** JIA exhibited greater sleep disruption than controls, including reduced NREM sleep, longer total sleep time, and increased wake time after sleep onset. HRV analyses in both time and frequency domains revealed significant differences between groups across all stages of sleep. In JIA patients, the standard deviation of the normal-to-normal interval during slow wave sleep (SWS) and total power across all sleep stages (*p* < 0.05) was reduced. In JIA patients, the standard deviation of the normal-to-normal interval during slow wave sleep and total power across all sleep stages were significantly reduced (*p* < 0.05). VLF power was also significantly lower in JIA patients across all sleep stages (*p* = 0.002), with pronounced reductions during N2 and SWS (*p* = 0.03 and *p* = 0.02, respectively). A group effect was observed for total power across all stages, mirroring the VLF findings. Additionally, group differences were detected in LF/HF ratio analyses, although values during N2, SWS, and REM sleep did not differ significantly between groups. Notably, the number of affected joints showed a moderate positive correlation with the parasympathetic HRV parameter. **Conclusions:** Patients with JIA exhibited sleep disruption and alterations in cardiovascular autonomic functioning during sleep. Reduced HRV across all sleep stages in these patients suggests underlying autonomic nervous dysfunction. Addressing sleep disturbances in patients with chronic pain may serve as an effective strategy for managing their cardiovascular risk.

## 1. Introduction

Juvenile idiopathic arthritis (JIA) encompasses a group of disorders of unknown etiology that affect children under 16 years, characterized by chronic joint inflammation lasting more than 6 weeks [1]. The autonomic nervous system modulation in childhood chronic inflammatory conditions, as in patients with JIA, has potential implications for long-term cardiovascular health [2]. Cardiac manifestations of varying severity have been reported in the progression of JIA, involving the pericardium, myocardium, endocardium, coronary vessels, valves, and conduction system. Early detection of subclinical cardiac involvement may help prevent cardiovascular morbidity and mortality [2,3].

Heart rate variability (HRV) analyses provide insights into cardiovascular health at any age [4]. HRV arises mostly from the interaction between the activities of sympathetic and parasympathetic tones, and it is typically measured through the RR interval (time between R waves) [4]. HRV reflects the dynamic balance between sympathetic and parasympathetic influences on cardiac function and serves as a biomarker for cardiovascular risk. It is regulated by afferent vagal inputs from the periphery and efferent projections from brain regions like the amygdala to the brainstem, modulating parasympathetic output to the heart [5]. Regarding heart rate variability origins, methods, and interpretive caveats, HRV can be assessed through RR interval analysis, typically recorded during wakefulness, for example using Bluetooth-connected sensors, as was conducted in a study with medical students [6].

Sympathetic activity increases heart rate, while parasympathetic input slows it, with imbalances linked to poor physiological and emotional regulation, especially under chronic stress [7]. While wakeful HRV is affected by stress and activity, sleep offers a stable condition to assess autonomic function, typically characterized by dominant vagal tone and reduced sympathetic activity, although phasic sympathetic bursts can occur and may even be protective for post-myocardial infarction [8].

The high frequency (HF) component of HRV is widely recognized as a marker of vagal activity, while the low-frequency (LF) component reflects both vagal and sympathetic influences. Low HRV is associated with an increase in cardiovascular risk, including mortality [9]. Additionally, the LF/HF ratio is commonly used to assess sympathovagal balance and its fluctuations during sleep [10,11]. The VLF component has been identified as a reliable marker of sleep recovery [12]. Changes in autonomic function across the sleep–wake cycle have been associated with the development of spontaneous hypertension [13].

Sleep expression follows a hierarchical process, with HRV patterns varying across different sleep stages. During NREM sleep, sympathetic activity decreases, while parasympathetic activity increases [12]. In slow wave sleep (SWS) and tonic REM sleep, there is an accentuation of the sympathetic–parasympathetic imbalance [14]. During phasic REM sleep, rapid and brief cycles of activation and inhibition of both parasympathetic and sympathetic tones are observed [4]. In rheumatic illnesses, autonomic dysfunction may arise, contributing to the initiation and perpetuation of the diseases [15]. Similarly, sleep disturbances in fibromyalgia have been linked to autonomic dysfunction, as evidenced by cardiopulmonary coupling evaluations [11]. The inflammatory response modulates autonomic nervous system (ANS) activity in both critical illness and chronic inflammatory conditions such as asthma. Proinflammatory markers, alongside ANS alterations, may serve as indicators of critical illness in the pediatric population. For instance, heart rate variability (HRV) data may enable earlier detection of sepsis, potentially leading to earlier therapeutic intervention, reduced duration of organ dysfunction, and lower mortality [16].

Cardiovascular involvement is a significant source of morbidity and mortality in patients with rheumatologic diseases, including JIA [17]. Previous studies have linked chronic pain related to inflammation and/or decreased range of motion in one or more joints, with sleep disruption in JIA patients [18,19]. This study aimed to investigate changes in cardiovascular autonomic function through HRV measures in youth with JIA in their active phase of the disease.

## 2. Methods

### 2.1. Procedure and Participants

We recruited 10 children (5 girls/5 boys) and teenagers with JIA and 10 age-, gender-, and Tanner stage group-matched control subjects from the community. All participants underwent monitoring following one night of habituation in the sleep laboratory. JIA patients met the American College of Rheumatology criteria for active polyarticular JIA [20]. Inclusion criteria included clinical arthritis and/or elevated erythrocyte sedimentation rate and/or reactive C-protein, indicating inflammation for at least 6 weeks. Exclusion criteria included neuropsychiatric disorders, acute pain from other conditions (assessed using a visual analog scale before sleep), clinical symptoms, or signs of sleep disorders. Control subjects were screened through clinical evaluation, sleep/wake questionnaires, and polysomnography.

In both groups, participants and their legal caregivers provided informed consent in accordance with the ethical board of Sao Paulo Federal University. Participation was entirely voluntary.

### 2.2. Recording and Sleep–Wake Conventional Analysis

Sleep/wake schedules and typical sleep time were recorded using one-week sleep logs. Participants were instructed to arrive at the sleep laboratory around 7 PM for a one-night adaptation to sleep monitoring, followed by the test night. Recordings took place during participants usual sleep time, with lights out based on the sleep logs. A minimum duration of 7 ½ hours of total sleep time was recorded using a computerized sleep system (Stellate System™, Harmonie 2.4; Montreal, Quebec, Canada) with a sampling frequency of 200 Hz/channel. The following variables were monitored: EEG (C3-A2, C4-A1, O1-A2, O2-A1), electrooculogram (right and left), electromyogram (chin and anterior tibial), one lead-electrocardiogram (modified V2 lead), respiration (via oral and nasal thermistors, microphone, thoracic, abdominal belts) and pulse oximetry (Ohmeda™, Helsinki, Finland). Evening and morning questionnaires were administered to assess the subjective quality of wake and sleep before the study in the laboratory.

Sleep stages and wakefulness were scored using standardized criteria [21]. Two sleep experts independently reviewed the analyses: sleep onset latency (defined as three consecutive epochs of stage (1)), total sleep time (TST), sleep efficiency (TST/total recording time), and time awake after sleep onset. Percentages of TST, NREM, and REM sleep stages were also tabulated. Arousals were identified based on short (≥3 s) EEG shifts, following the American Sleep Disorders Association arousal definition [22]. The arousal events were scored according to adult criteria, excluding delta activity, and were defined as abrupt shifts in EEG toward fast activity such as 8–13 Hz (alpha) or >16 Hz (beta) (but not spindles) lasting at least 3 s. In REM sleep, an increase in submental EMG amplitude was required to score an arousal event. A minimum interval of 10 s of continuous sleep was necessary to score any event. The arousal index was derived from these tabulations.

### 2.3. Heart Rate Variability (HRV) Analysis During Sleep

A thorough manual review of the full recording was performed to exclude artifacts and arrhythmias from the analysis of heart rate variability (HRV), which was derived from cardiac sensors such as electrocardiography in the standard D2 derivation. In accordance with the Task Force of the European Society of Cardiology [23], a minimum sampling frequency of 250 Hz was used for HRV analysis. The central 5 min segment of the longest artifact-free sleep stages from the second sleep cycle was selected for analysis. HRV was assessed using time-domain and frequency-domain methods. Frequency-domain (power spectral density) analysis characterizes the heart rate oscillations at different frequencies and amplitudes in short-term (5-min) recordings, while time-domain analyses evaluate HRV over longer periods. The HRV analysis was performed without knowledge of the patient’s clinical condition.

### 2.4. Time Domain Analysis

During continuous ECG recording, each QRS complex was detected, and the normal-to-normal (NN) intervals between each R-R wave were calculated. Five time-domain indexes were derived: the standard deviation of all normal-to-normal intervals (SDNN), the mean of the standard deviation of the 5 min NN intervals over the entire recording (SDNN index), the root mean square of the difference between successive NN intervals (RMS), and the proportion of adjacent normal NN intervals differing by >50 ms, referred to as pNN50 [23,24].

### 2.5. Frequency Domain Analysis

Five consecutive minutes of stable artifact-free ECG data were collected during sleep stages N2, SWS, and REM sleep. Spectral indices for HRV were computed using fast Fourier transforms (FFT) of the detrended instantaneous heart rate (IHR), derived from resampling the RR intervals [23]. Power densities for the very low-frequency (VLF, 0.0033–0.04 Hz), low-frequency (LF, 0.04–0.15 Hz), and high-frequency (HF, 0.15–0.4 Hz) components were calculated by integrating the power spectral density in the respective frequency bands for each sleep stage. The normalized power spectra LF/HF ratio was also computed. Results were expressed in ms^2^/HzEq [23].

### 2.6. Statistical Analysis

Central tendency measures were reported as mean and standard deviation. After assessing the normality of distribution, Friedman tests were used to compare HRV parameters across NREM sleep stages within each group. Data were tabulated and analyzed using SPSS Statistics for Windows, Version 22.0, IBM Corp. (Armonk, NY, USA). The significance level of *p* < 0.05 was applied.

## 3. Results

The characteristics of the samples were according to the recruitment methods, including 10 children with JIA (mean of age of 12 ± 3 years; Tanner stage: 3) and 10 age-, gender-, and Tanner stage group-matched control subjects from the community (mean of age of 13 ± 2 years; Tanner stage: 3). We documented the time of disease, the number of impaired joints, and the erythrocyte sedimentation rate (see Table 1).

### 3.1. Sleep Macroarchitecture

Table 2 displays the results of the nocturnal sleep analysis. Children with JIA exhibited reduced SWS increased total sleep time and longer wake time after sleep onset (WASO). Additionally, children with JIA further showed greater sleep fragmentation, characterized by EEG arousals compared to healthy controls.

### 3.2. HRV Analyses

Table 3 shows the differences in HRV time and frequency domains. JIA patients had significantly lower SDNN and VLF during SWS. In JIA patients, the standard deviation of the normal-to-normal interval during SWS and total power (TP in all sleep stages was reduced inpatients with JIA (See Table 3). The VLF was decreased in all sleep stages for JIA patients, with a marked reduction during N2 sleep stage and SWS compared to the control group. We also found changes in the TP when we compared JIA and the control group in all stages (similar with VLF) (see Supplementary Data). Moreover, we found another group effect in the LF/HF analyses where the N2 sleep stage, SWS, and REM sleep were similar between JIA and the control group. The TP was significantly lower in JIA throughout all sleep stages. In terms of NN50 and pNN%, minutes of SWS were significantly lower compared to N2. On the other hand, we observed VLF in REM sleep that was higher than in N2 and REM sleep higher than in SWS, and we also found changes in the TP comparing the JIA and control group, in which N2 was higher than REM sleep. Moreover, we found another group effect in the LF/HF analyses in which the N2 and SWS were similar between the JIA and control group, despite the difference in REM sleep.

## 4. Discussion

This pilot study is the first to investigate the potential role of reduced HRV during sleep in patients with juvenile idiopathic arthritis. Our findings demonstrate autonomic imbalance and sympathetic overflow across all sleep stages, which are associated with disease severity. The relationship between the autonomic nervous system and the subjective experience of pain suggests that higher parasympathetic activity may enhance self-regulation and pain inhibition capacities. HRV analyses have been linked to recovery processes in humans [2]. We observed a decrease in VLF, indicative of a sympathovagal imbalance. Previous studies have shown that the renin–an giotensin system is associated with the VLF band, with a stronger correlation to cardiovascular disease prognosis, metabolic syndromes, and all-cause mortality following traumatic brain injury compared to other HRV components. Low VLF power has consistently been linked to chronic inflammation, and the nocturnal VLF band may serve as a predictor of infection following acute stroke [2].

Over the recent decades, the number of studies examining autonomic dysfunction, assessed via HRV in the pediatric population has increased, particularly in inflammatory conditions, pain processes, and atherosclerosis associated with rheumatoid arthritis [25,26,27]. Abnormal HRV, characterized by high sympathetic tone and reduced HRV, has been linked to cardiovascular morbidity in adults with obstructive sleep apnea (OSA) [28,29] and other chronic medical conditions [30]. In fact, Martín-Montero et al. (2021) [28] proposed that HRV could serve as a potential biomarker for OSA treatment, based on a multicenter study involving over 400 children. Our findings in patients with JIA showed changes in the time-domain analysis of HRV that correlated with the number of impaired joints. The pNN50 parameter, primarily reflecting parasympathetic activity [31], suggests that chronic arthritis may disrupt autonomic balance. Vascular changes, such as delayed brachial artery dilation in response to hyperemic stress, sympathetic overactivity, and elevated blood pressure [28], may act as cardiovascular sequelae in other sleep disorders such as sleep-disordered breathing in childhood. We hypothesize that early disruptions in parasympathetic activity may exacerbate cardiovascular risk in patients with JIA. HRV analyses during sleep could, therefore, be a valuable tool for monitoring these patients.

We observed early changes in HRV in youth with IJA, both in the time-domain and the frequency-domain, across each sleep stage. The autonomic nervous system balance, depending on the sleep stage, results from the interaction between parasympathetic and sympathetic activity, which may be altered due to peripheral disturbances that may trigger central nervous system activation. The strong coupling between cardiac and EEG activity suggests common pathways within the central autonomic network that regulate cardiovascular responses [32,33] and neuronal activity [34]. These interactions could be modulated by noradrenergic neurotransmitters, which influence ‘REM-off’ and ‘REM-on’ factors [35] or act as sleep-promoting and sleep-wake transition regulators [36,37]. Chronic sleep disruption could lead to an imbalance in the autonomic system, potentially increasing cardiovascular mortality in patients with chronic arthritis. Additionally, cardiovascular and cognitive aging have been connected in the context of multimorbidity [38]. In this context, distinct branches of the autonomic system have been linked to cognitive decline: parasympathetic activity (PNS) is associated with healthy cognitive aging, while sympathetic activity (ANS) is linked to accelerated cognitive decline [39,40]. Therefore, HRV during sleep across the lifespan could serve as an important biological biomarker for cognitive decline.

The sympathetic nervous system and the vagal nerve of the parasympathetic nervous system play key roles in regulating cardiac variability. High HRV is typically associated with the cardiac system’s ability to adapt to acute stress (e.g., surgery, exercise) or chronic diseases, while low HRV is linked to cardiovascular impairment [27]. In our study, we observed a correlation between inflammatory markers and HRV parameters (LF and erythrocyte sedimentation rate and pNN50 with the number of impaired joints); these correlation could be explained by a cholinergic anti-inflammatory mechanism, suggesting that the nervous system regulates the inflammatory response as part of a compensatory process [41,42] Moreover, HRV parameters may serve as a predictive tool for sepsis risk in patients with chronic illnesses, as decreased HRV has been associated with a worse prognosis [41] Our findings showed a decrease in TP in young patients with JIA compared to controls. In addition, HRV parameters such as LF power were negatively correlated with interleukin-6 levels [42]. Previous studies have found that sepsis survivors exhibit higher HRV parameters, such as SDNN, than nonsurvivors [43]. While we hypothesize that HRV during sleep could be an early marker of morbidity, we also found that the level of systemic inflammation in youth with sleep-disorder breathing did not influence the balance between the sympathetic and parasympathetic systems during sleep [44]. However, a significant gap remains in understanding the interaction between autonomic modulation, sleep instability, and proinflammatory biomarkers of patients with JIA. Asthmatic children exhibit an increase in the high-frequency (HF) component of heart rate variability (HRV), indicating enhanced parasympathetic activity [45]. Asthmatic children exhibit an increase in the high-frequency (HF) component of heart rate variability (HRV), indicating enhanced parasympathetic activity [46]. Notably, parasympathetic modulation differs between asthma and juvenile idiopathic arthritis (JIA) [47]. The balance between sympathetic and parasympathetic activity reflects homeostatic regulation and is influenced by both physical activity and circadian rhythms. In contrast, our findings revealed alterations in the very-low-frequency (VLF) component, which may reflect recovery processes specific to patients with JIA.

According to Thomas et al. (2010) [14], sleep should be evaluated as part of a coupling process, that involves the respiratory and cardiovascular systems. These researchers developed a fully automated surface electrocardiogram (ECG)-derived sleep physiology estimator, which quantitatively analyzes cardiopulmonary coupling from a single-lead ECG. This method combines the mechanical and autonomic effects of physiological and periodic breathing on ECG parameters related to heart rate variability and the QRS electrical axis [14]. The frequency-domain serves as a tool for analyzing the balance between sympathetic and parasympathetic systems, as well as the recovery processes during sleep. In our study, we observed changes in LF/HF ratio during SWS and REM sleep in children with JIA. The LF/HF ratio may reflect central, baroreflex, or cellular membrane responses to different stimuli during severe stress [48]. Some studies have found that the altered LF/HF ratio during sleep can serve as an indicator of autonomic nervous dysfunction in children, especially when correlated with the severity of OSA, though not with age [44]. Our findings related to REM sleep suggest that this sleep stage might serve as a potential marker of sleep disruption and brain maturation in patients with JIA [48]. Additionally, we observed low VLF in NREM, particularly during SWS. which could be a marker for better cardiovascular prognosis in youth with chronic arthritis. The VLF band reflects sympathovagal balance and has unique characteristics. Studies have shown that the renin–angiotensin system is associated with the VLF band and plays a stronger role in cardiovascular disease prognosis, metabolic syndromes, and all-cause mortality after traumatic brain injury than other HRV components. Furthermore, low VLF power has been linked to increased chronic inflammation, and the nocturnal VLF band may predict infection after acute stroke [49]. The induction of proinflammatory activity in humans is known to decrease HRV parameters [50], supporting the bidirectional relationship between inflammation and autonomic nervous systems output. The interaction of comorbidities, particularly sleep conditions in severe illness, may explain some of the poor outcomes seen in patients receiving intensive care.

The homeostatic process influences the expression of HRV during the night and can lead to variations in HRV measurements across each sleep cycle. In our analysis, we focused on the second cycle of sleep. Despite the small sample, we believe that including HRV data from the wakefulness period before sleep could provide additional insights into our findings. Furthermore, we excluded sleep-disordered breathing in all participants, as breathing difficulties could potentially influence HRV. While some studies suggest that the relationship between cardiovascular processes and sleep may limit the ability of HRV to accurately assess the sympathovagal balance [51,52,53]; we did not observe any changes in vascular inflammation using HRV measurements. It is also important to note that heart rate regulation has opposing effects on sympathetic and vagal outflows with additional modulation factors influencing the vasculature [54].

The hierarchical process of sleep is closely connected to heart rate regulation. The homeostatic process of sleep is linked to the alostatic load of beat-to-beat oscillations, and changes in breathing patterns. However, SWS is associated with thalamocortical oscillations, where the thalamus acts as a gate, and vagal stimulation can reduce the stability of SWS. There is both a long-term homeostatic process and acute changes resulting from sleep disorders. This diminished recovery process is evident in conditions like migraine, which is associated with hypothalamic dysfunctions, particularly involving the orexinergic system. This dysfunction likely contributes to the connection between pain, reduced arousability, and neurovascular headaches [55,56]. Additionally, SWS plays a role in synaptic plasticity. Our findings suggest a delay in sleep recovery in youth with JIA, as indicated by the decrease in VLF during NREM sleep, particularly in SWS. The VLF may reflect the homeostatic balance attempting to recover from a sympathovagal imbalance. Disease duration may further exacerbate HRV alterations, particularly in the context of chronic pain [7,12]. Additionally, reduced parasympathetic activity has been associated with increased cardiovascular risk [57,58]. These findings underscore the potential of HRV as an early biomarker and highlight the need for longitudinal studies to confirm its prognostic value in JIA.

We observed several differences in HVR measures between patients with JIA and controls. In patients with JIA, biomarkers have been linked to sleep fragmentation, a common sleep complaint [59,60]. Despite our patients with JIA reporting poor sleep, we did not find significant differences in the time spent in each sleep stage when comparing patients to controls. In fact, total sleep time was longer in patients with JIA, suggesting increased sleep pressure associated with chronic illness. This study has some limitations. First, we did not control for the potential effects of medication on HRV parameters, despite the known influence of pharmacological treatments on autonomic function in patients with JIA. Future studies should account for medication use when examining HRV and sleep architecture in this population. Second, physical activity levels were not objectively monitored (e.g., via actigraphy), and circadian rhythm assessments (such as chronotype) were not conducted. Given that sleep patterns can affect autonomic regulation and modulate the interplay between sleep, HRV, and JIA, these factors warrant consideration. Previous research has shown that moderate-intensity exercise can improve sleep quality and restore cardiac autonomic function [61,62]. However, the impact of prevention of the development of cardiovascular involvement in children is still unclear, and there is a need to assess the risk of cardiovascular disease in patients with JIA, and it requires long-term follow-up in terms of lifestyle changes, eating habits [58], and sleep patterns. Future studies should investigate exercise as a potential intervention to mitigate HRV alterations in JIA.

In conclusion, HRV analyses across all sleep stages can serve as a crucial bioalgorithm for detecting autonomic dysfunction in pediatric patients with JIA. Recognizing early imbalances between sympathetic and parasympathetic systems likely will change the follow-up in chronic cases of these conditions that may have both short- and long-term consequences for children with health conditions, particularly those experiencing chronic pain during sleep and wakefulness.

## Figures and Tables

**Table 1:** Characteristics of the sample.

	Controls (n = 10)	JIA (n = 10)
Age (years)	12.3 ± 2.5	12.9 ± 2.2
Girls:Boys	5:5	5:5
Tanner scale	Stage 3	Stage 3
Impaired joints (N)	0	11.7 ± 11
Erythrocyte sedimentation rate	Not detected	37.6 ± 27.4

n = number of subjects; N = number of impaired joints.

**Table 2:** Comparison of macrostructure and arousal index between controls and JIA patients.

	**Controls** **(n = 10)**	**JIA** **(n = 10)**	**Mann–Whitney U Test**	***p*** **-Value**
SL (min)	11.1 ± 7.8	19.6 ± 17.0	39.5	0.4
TST (min)	463.1 ± 36.6	420.7 ± 55.3	28	0.1
WASO (min)	21.1 ± 12.7	47.3 ± 38.1	28.5	0.1
SE (%)	95.4 ± 2.9	90.1 ± 7.1	26.5	0.1
N1 (min)	20.5 ± 9.9	17.7 ± 9.4	42	0.6
N2 (min)	266.8 ± 45.0	240.9 ± 37.7	30	0.1
SWS (min)	89.1 ± 26.8	77.8 ± 27.1	44	0.7
REM (min)	87.2 ± 22.5	83.5 ± 28.0	46.5	0.8
Arousal index	2.7 ± 1.2 *	10.7 ± 5.6	13	0.01

Data presented in mean ± standard deviations. SL: sleep latency; TST: total sleep time; SE: sleep efficiency; N1, stage N1 sleep; N2, stage N2 sleep; SWS: slow wave sleep NREM. * *p* < 0.01.

**Table 3:** Sleep stages and HRV parameters within each group by Friedman Anova test.

	**JIA**			**Friedman Anova**		**Control**			**Friedman Anova**	
	**N2**	**SWS**	**REM**	**X2(10.2)=**	***p*** **-Value**	**N2**	**SWS**	**REM**	**X2(10.2)=**	***p*** **-Value**
RRi	805.2 ± 140.4	789.0 ± 148.8	779.0 ± 166.3	7.2	**0.03**	901.9 ± 142.8	901.9 ± 131.5	860.6 ± 119.2	1.90	0.39
SDNN	62.8 ± 48.2	47.0 ± 38.5	72.0 ± 40.4	8.6	0.01	94.3 ± 48.6	94.6 ± 75.2	111.0 ± 53.0	1.4	0.50
RMSSD	74.3 ± 60.8	59.0 ± 56.8	63.3 ± 58.9	5.06	0.08	98.5 ± 63.5	109.8 ± 96.9	99.8 ± 71.9	0.15	0.93
SDSD	46.6 ± 40.4	35.4 ± 34.6	47.8 ± 42.4	4.05	0.13	68.6 ± 49.3	80.4 ± 83.0	80.6 ± 59.2	1.4	0.50
NN50	121.4 ± 108.6	90.6 ± 104.3	84.8 ± 90.96	0.67	0.72	156.7 ± 55.2	149.5 ± 75.4	124.2 ± 52.33	10.4	0.005
pNN50	36.5 ± 34.2	27.9 ± 33.5	25.7 ± 30.2	0.15	0.93	49.1 ± 20.9	47.1 ± 25.9	37.5 ± 18.0	9.8	0.008
VLF	1407.8 ± 1552.3	852.6 ± 551.8	5872.3 ± 2786.5	12.2	0.002	4290.3 ± 4775.6	2494.5 ± 2657.7	6563.6 ± 3417.5	4.2	0.13
LF	2100.1 ± 1578.2	1734.2 ± 1328.0	2155.1 ± 1422.5	3.2	0.02	3183.1 ± 1841.9	2544.2 ± 2210.9	2779.0 ± 1098.1	7.2	0.03
HF	3635.1 ± 2224.3	2706.3 ± 1678.8	2031.3 ± 1545.0	11.4	0.003	3917.1 ± 2099.6	3727.7 ± 2552.6	3065.6 ± 2282.0	1.4	0.50
TP	7322.3 ± 4197.1	5433.3 ± 2802.2	10338.0 ± 2856.6	9.8	0.008	11703.7 ± 5298.4	9108.5 ± 5251.9	12995.2 ± 2920.9	2.6	0.27
LF/HF	1.1 ± 1.6	1.0 ± 1.3	2.1 ± 3.1	7.4	0.03	1.2 ± 1.2	0.8 ± 0.6	1.4 ± 1.4	7.2	0.03

Legend: Data were presented as mean ± standard deviation. Difference in HRV parameters, according to the interaction between two factors (sleep stages and groups). RRi, the interval between beat to beat (RR); SDNN, standard deviation of NN intervals for period of interest; RMSSD, root mean square of successive differences of NN intervals for period of interest; SDSD, standard deviation of successive differences; NN50, NN intervals > 50 ms different from previous (NN) for period of interest; pNN50, percentage of NN intervals > 50 ms different from previous (NN) for period of interest root mean square of successive differences of NN intervals for period of interest; VLF, very low frequency; LF, low frequency; HF, high frequency; TP, total power; LF/HF, average over 5 min periods or less that are purported to reflect sympathetic nervous system per parasympathetic nervous.

## Data Availability

All data can be assessed by the first author, and supporting reported results can be found directly with the first author. The data are not publicly available due to ethical reasons.

## Supplementary Materials

The following supporting information can be downloaded at: https://www.mdpi.com/article/10.3390/brainsci15060648/s1, Figure S1: Example of a representative hypnogram. Figure S2: Example of 5minutes epoch without artifacts. Table S1: Difference in HRV parameters during N2 sleep stage between two factors (sleep stages and groups). Table S2: Difference in HRV parameters during slow wave sleep (SWS) sleep stage between two factors (sleep stages and groups). Table S3: Difference in HRV parameters during REM sleep stage between two factors (sleep stages and groups).
