# Supplementary material for: Sleep in Juvenile Idiopathic Arthritis: An Exploratory Investigation of Heart Rate Variability
Source: Brain Sci. 2025 Jun 17;15(6):648. doi: 10.3390/brainsci15060648 (PMC12191250; doi:10.3390/brainsci15060648)
Supplement: Supplementary file 1 [file brainsci-15-00648-s001.zip › brainsci-3651298-supplementary.pdf]

Figure S1:

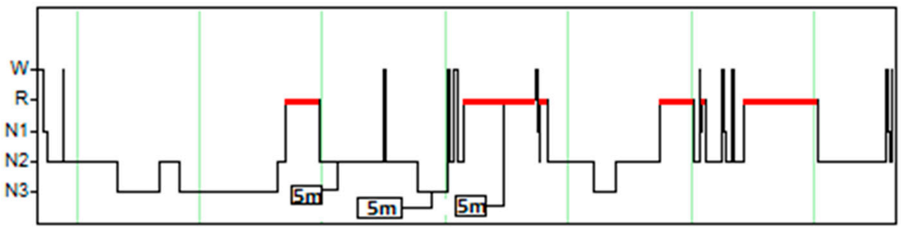

Legend: Figure S1 has a representative full-night hyponograma from one of the subjects in your study. The vertical lines with the mention of 5m indicates the time points at which 5 minute segments of stable sleep were extracted for heart rate variability (HRV) analyses.
In N2, N3, and REM sleep stages, sleep stages are represented on the y-axis, with time in hours on the x axis. The selection prioritized artifact-free, stable epochs to ensure reliable HRV assessment.

Figure S2:

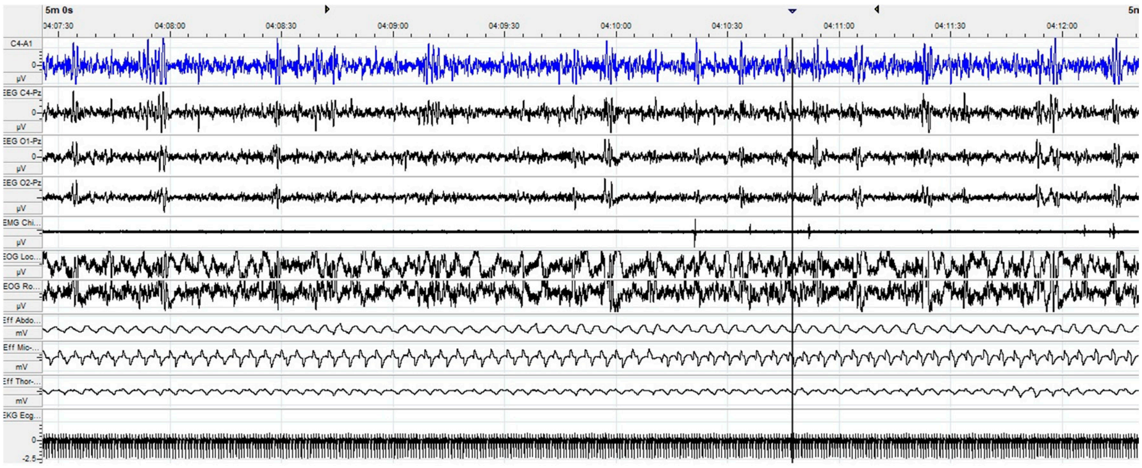

Legend: Figure S2 shows an example of a 5 minute epoch of stable sleep used for heart rate variability (HRV) analysis. This segment corresponds to one of the selected periods of undisturbed NREM sleep, meeting criteria for signal stability and artifact-free recording.

We included graphs about the differences between groups in the supplementary section. The legend for all graphs shows that all data about the differences between groups were present in the description; the whiskers are the minimum and the maximum; the cross is the mean; the line in the box is median; the bottom of the box is lower quartile, and upper line of the box is upper quartile. See them below:

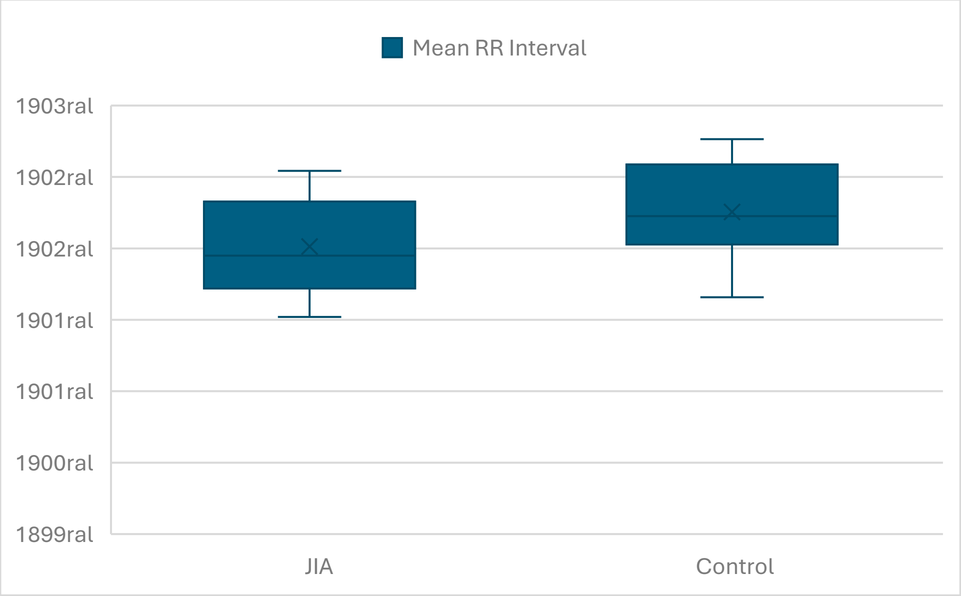

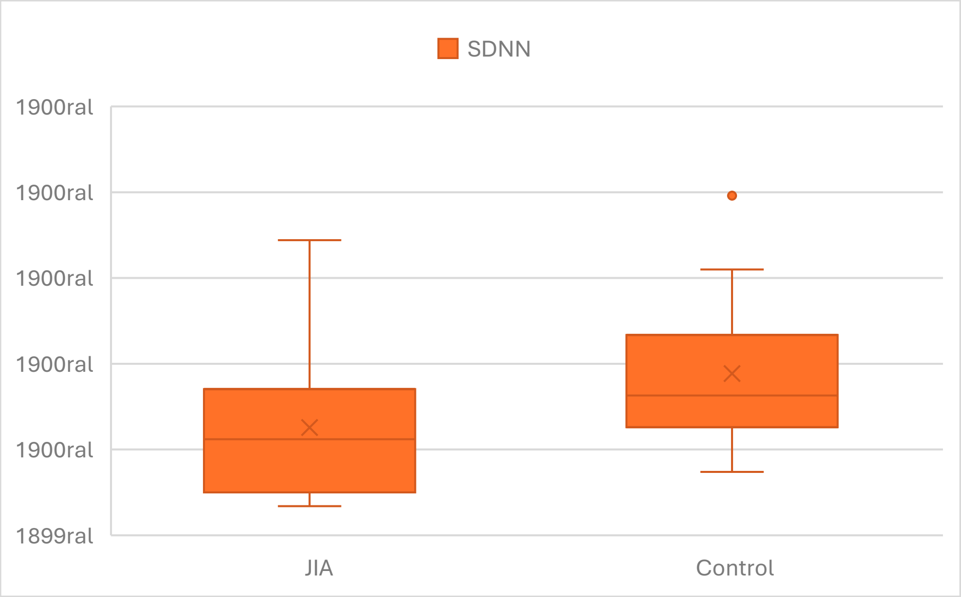

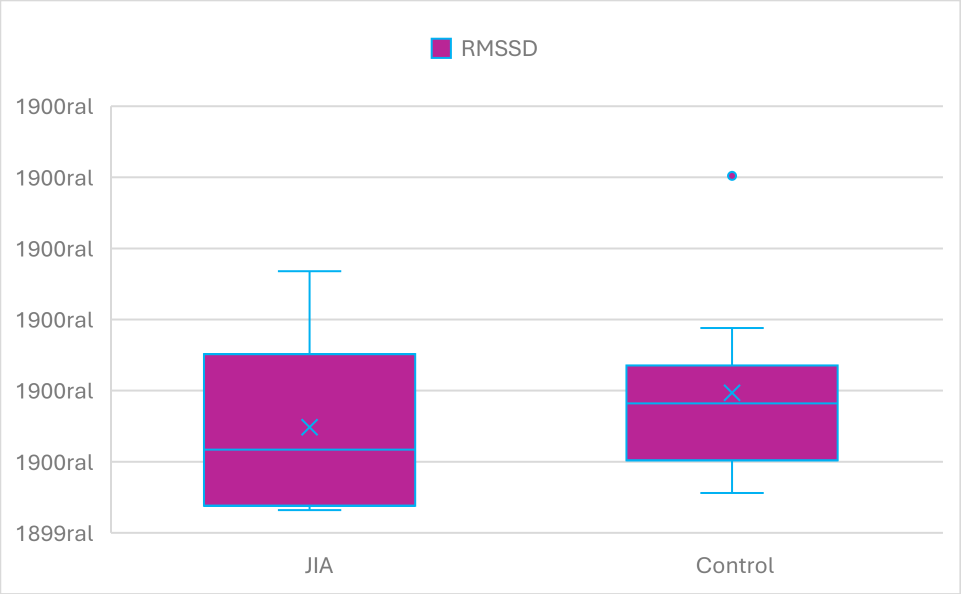
RMSSD
1900ral
1900ral
1900ral
1900ral
1900ral
1900ral
1899ral
JIA
Control

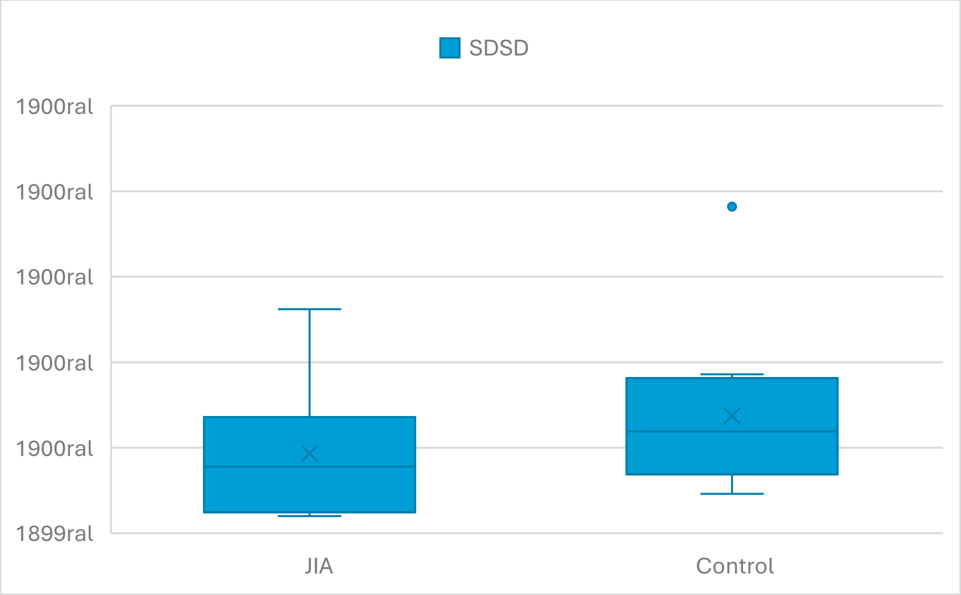
SDSD
1900ral
1900ral
1900ral
1900ral
1900ral
1899ral
JIA
Control

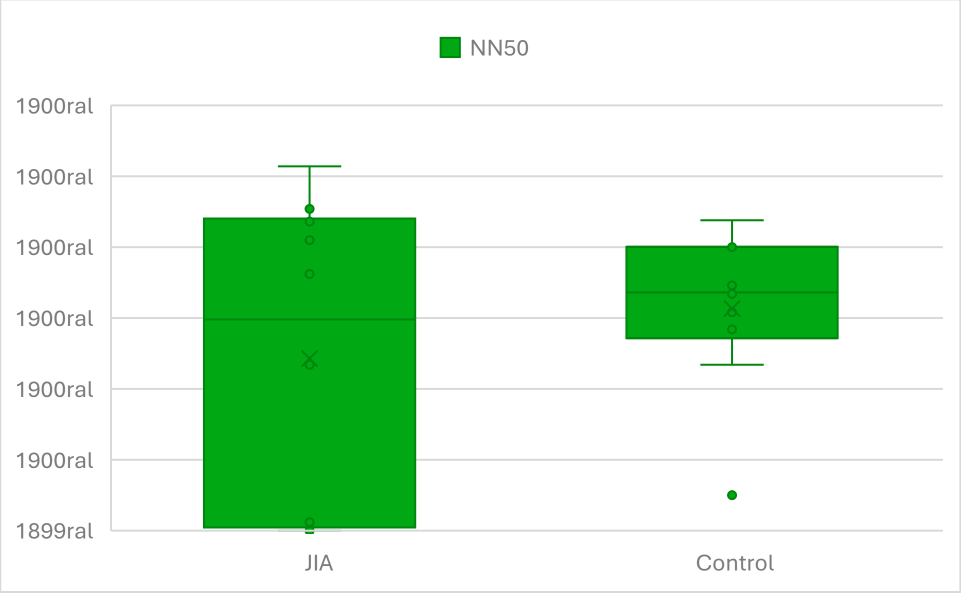
NN50
1900ral
1900ral
1900ral
1900ral
1900ral
1900ral
1899ral
JIA
Control

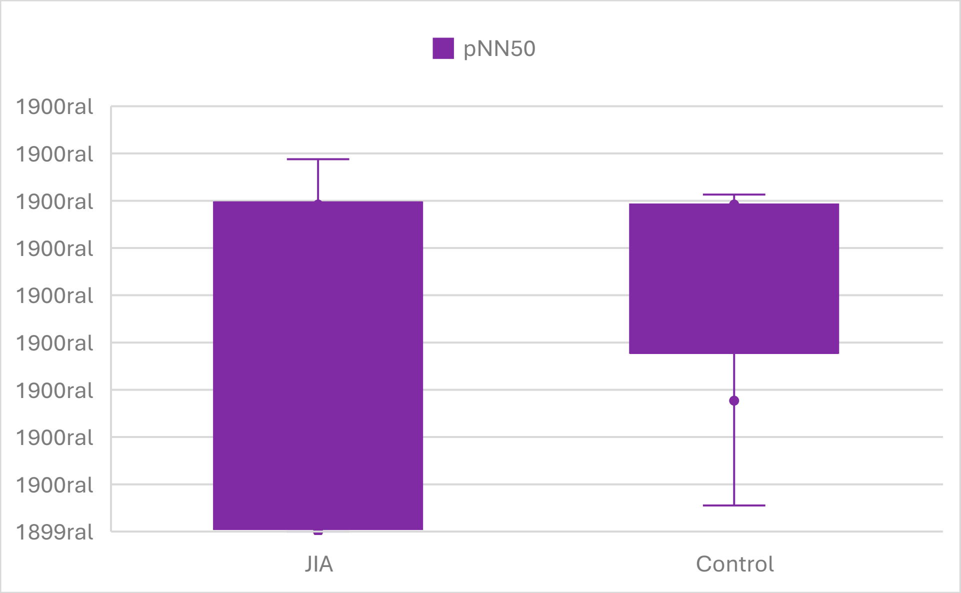
pNN50
1900ral
1900ral
1900ral
1900ral
1900ral
1900ral
1900ral
1900ral
1900ral
1899ral
JIA
Control

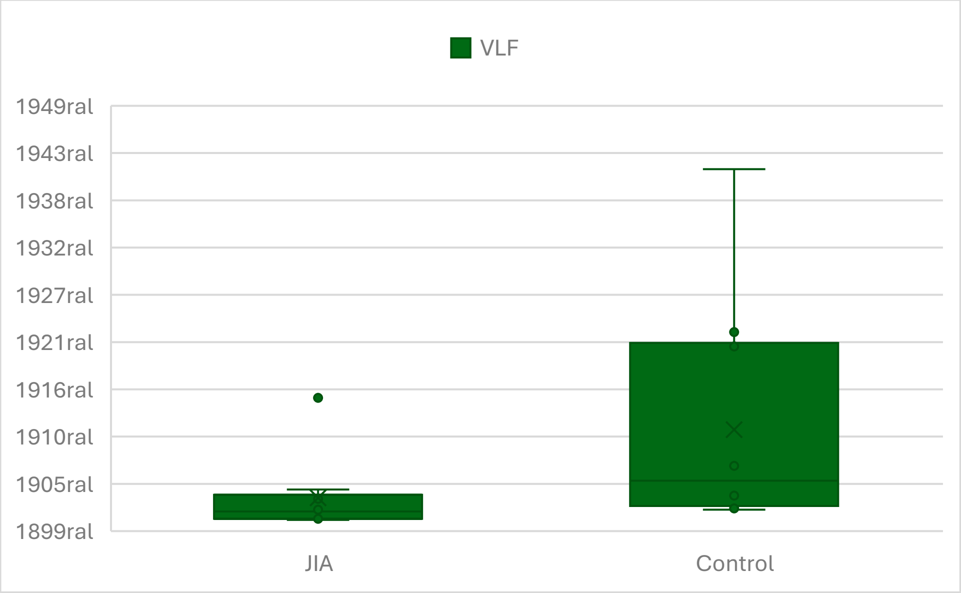
VLF
1949ral
1943ral
1938ral
1932ral
1927ral
1921ral
1916ral
1910ral
1905ral
1899ral
JIA
Control

We also included the data in the tables across each stage.

Table S1: See below the HRV parameters in N2

	JIA		control			
	mean	sd	mean	sd	U	p-value
Mean RR I	805.200	140.445	901.90	142.802	32.00000	0.190316
SDNN	62.800	48.182	94.30	48.569	29.50000	0.123005
RMSSD	74.300	60.826	98.50	63.519	37.00000	0.352681
SDSD	46.600	40.371	68.60	49.329	33.00000	0.217563
NN50	121.400	108.649	156.70	55.162	46.50000	0.795936
pNN50	36.470	34.191	49.06	20.889	41.50000	0.528849
VLF	1407.800	1552.341	4290.30	4775.586	21.00000	0.028806
LF	2100.100	1578.193	3183.10	1841.925	31.00000	0.165494
HF	3635.100	2224.285	3917.10	2099.645	42.00000	0.578742
TP	7322.300	4197.093	11703.70	5298.390	22.00000	0.035463
LF/HF	1.122	1.556	1.25	1.178	38.00000	0.393048

Table S2: See below the HRV parameters in SWS

	JIA		control			
	mean	sd	mean	sd	U	p-value
Mean RR I	798.000	148.846	901.900	131.489	27.50000	0.089210
SDNN	47.000	38.511	94.600	75.202	20.00000	0.023231
RMSSD	59.000	56.849	109.800	96.873	27.00000	0.089210
SDSD	35.400	34.590	80.400	83.038	24.50000	0.052426
NN50	90.600	104.270	149.500	75.449	32.50000	0.190316
pNN50	27.900	33.461	47.110	25.919	31.50000	0.165494
VLF	852.600	551.784	2494.500	2657.690	17.50000	0.011496
LF	1734.200	1328.017	2544.200	2210.878	38.50000	0.393048
HF	2706.300	1678.819	3727.700	2552.620	41.50000	0.528849
TP	5433.300	2802.163	9108.500	5251.891	23.50000	0.043257
LF/HF	0.964	1.291	0.843	0.620	44.50000	0.684211

Table S3. See below the HRV parameters in REM sleep

	JIA		control			
	mean	sd	mean	sd	U	p-value
Mean RR I	779	166.268	860.60	119.152	31.5	0.165494
SDNN	71.6	40.368	111.20	52.978	22.5	0.035463
RMSSD	63.3	58.912	99.80	71.893	32.5	0.190316
SDSD	47.8	42.373	80.60	59.212	29.5	0.123005
NN50	84.8	90.965	124.20	52.330	36.5	0.314999
pNN50	25.69	30.180	37.54	17.950	31.5	0.165494
VLF	5872.3	2786.506	6563.60	3417.465	48.5	0.911797
LF	2155.1	1422.516	2779.00	1098.146	25.5	0.063013
HF	2031.3	1544.993	3065.60	2281.954	35.5	0.279861
TP	10338	2856.608	12995.20	2920.880	22.5	0.035463
LF/HF	2.08	3.054	1.42	1.353	48.5	0.911797
